# The psychological relevance and clinical impact of early screening and psychotherapeutic intervention in postnatal depression

**DOI:** 10.1192/j.eurpsy.2026.11392

**Published:** 2026-07-31

**Authors:** C. V. Valentina

**Affiliations:** Department of Mental Health, Medical Psychology and Psychotherapy, Nicolae Testemițanu State University of Medicine and Pharmacy, Chisinau, Moldova, Republic of

## Abstract

**Introduction:**

Postnatal depression (PND) affects up to one in five mothers and has profound effects on maternal mental health, infant development, and family functioning. Despite growing awareness, systematic screening and early psychotherapeutic support remain inconsistent across Europe. Psychotherapists play a vital role in early detection and the provision of accessible, non-stigmatizing care. This review synthesizes evidence on the psychological mechanisms and clinical benefits of early psychotherapeutic intervention, following PRISMA 2020 guidance.

**Objectives:**

The review aimed to examine key psychological factors underlying PND—emotional dysregulation, identity transition, and attachment disruption—and to evaluate the clinical impact of early screening and psychotherapeutic engagement within the first six months postpartum. A secondary aim was to assess how structured screening pathways enhance maternal well-being and relational outcomes.

**Methods:**

A comprehensive systematic search was performed in six major databases—MEDLINE, Embase, PsycINFO, Web of Science, Cochrane Library, and Scopus—encompassing all publications available until August 2025, without language restrictions. Interventions involved early screening and psychotherapy (CBT, IPT, attachment-based, or telepsychotherapy). Randomized, quasi-experimental, and mixed-methods studies were included. Risk of bias was assessed using Cochrane RoB 2, ROBINS-I, and CASP; evidence certainty was rated with GRADE. Due to heterogeneity, results were synthesized narratively.

**Results:**

Across studies, early screening followed by psychotherapeutic intervention significantly reduced depressive symptoms and improved maternal–infant bonding compared with usual care. Interventions initiated within six months postpartum and integrating cognitive-behavioral or relational components showed the most consistent benefits. Participants reported increased emotional regulation, self-compassion, and improved partner communication. Telepsychotherapy achieved similar outcomes to in-person formats, enhancing accessibility. Despite variability in study design and follow-up, the direction of evidence remained strongly positive.

**Conclusions:**

This review demonstrates that early detection and timely psychotherapy are key to effective management of postnatal depression. Systematic screening linked to accessible, evidence-based psychotherapeutic care reduces symptoms and strengthens maternal–infant relationships. Incorporating validated tools such as the EPDS within perinatal services should become standard practice. Further studies should evaluate long-term outcomes and optimize digital and hybrid care models to expand access and continuity of postpartum mental health support.

**Disclosure of Interest:**

None Declared

